# NRBP1 promotes malignant phenotypes of glioblastoma by regulating PI3K/Akt activation

**DOI:** 10.1002/cam4.70100

**Published:** 2024-08-16

**Authors:** Anli Zhang, Shichao Peng, Sibai Sun, Shan Ye, Ye Zhao, Qiang Wu

**Affiliations:** ^1^ Department of Pathology The Second Affiliated Hospital of Anhui Medical University Hefei China; ^2^ Division of Life Sciences and Medicine, Department of Pathology, The First Affiliated Hospital of USTC University of Science and Technology of China Hefei China; ^3^ Teaching and Research Section of Nuclear Medicine, School of Basic Medical Sciences Anhui Medical University Hefei China

**Keywords:** epithelial‐mesenchymal transition, glioblastoma, nuclear receptor‐binding protein 1, PI3K/Akt signaling pathway, pseudokinase

## Abstract

**Objectives:**

Glioblastoma (GBM) is the most aggressive of intracranial gliomas. Despite the maximal treatment intervention, the median survival rate is still about 14–16 months. Nuclear receptor‐binding protein 1 (NRBP1) has a potential growth‐promoting role on biology function of cells. In this study, we investigated whether NRBP1 promotes GBM malignant phenotypes and the potential mechanisms.

**Methods:**

The correlation between NRBP1 and glioma grade, prognosis in TCGA/CGGA databases and our clinical data were analyzed. Next, we conducted knockout and overexpression of NRBP1 on GBM cells to verify that NRBP1 promoted cell proliferation, invasion, and migration in vitro and in vivo. Finally, we detected the impact of NRBP1 on PI3K/Akt signaling pathway and EMT.

**Results:**

There was a correlation between elevated NRBP1 expression and advanced stage glioma, as well as decreased overall and disease‐free survival. The suppression of proliferation, invasion, and migration of tumor cells was observed upon NRBP1 knockout, and in vitro studies also demonstrated the induction of apoptotic cell death. Whereas, its overexpression is associated with high multiplication rate, migration, invasion, and apoptotic escape. GO enrichment and KEGG analysis revealed that NRBP1 regulated differentially expressed gene clusters are involved in PI3K/Akt signaling pathway, as well as EMT mediated by this pathway. Moreover, the effects of NRBP1 knockdown and overexpression on GBM were mitigated by MK‐2206 and SC79, both of which respectively function as an inhibitor and an activator of the PI3K/Akt signaling pathway. Similarly, the suppression of NRBP1 led to a decrease in tumor growth, whereas its overexpression promoted tumor growth in a mouse model.

**Conclusions:**

This study shows that NRBP1 promotes malignant phenotypes in GBM by activating PI3K/Akt pathway. Hence, it can function as both a predictive indicator and a new target for therapies in GBM treatment.

## 1 INTRODUCTION

Glioblastoma (GBM), comprising 12%–15% of malignant tumors within the central nervous system (CNS), represents one of the most prevalent diagnoses among adult CNS tumors.^1^ Over recent decades, even with the implementation of comprehensive multimodal therapies including surgical resection, chemotherapy, and radiotherapy, the prognosis for GBM remains exceedingly poor.^2^ The latest research indicates that nanomedicine holds immense potential for treating malignant GBM, with nanoparticles aiding in overcoming common drug resistance mechanisms observed in this type of tumor. By directly delivering drugs to tumor cells and potentially targeting multiple pathways involved in drug resistance, it may improve treatment efficacy.^3^ The persistent presence of aggressive characteristics, including high invasiveness, resistance to apoptosis, and robust proliferation, in GBM is closely linked to treatment failures and the recurrence of lesions.^4^, ^5^ Nevertheless, the specific mechanisms underlying the malignant phenotype of GBM have not been thoroughly explored.^6^ Hence, the mechanisms responsible for tumor cell proliferation, invasion, and apoptosis, as well as reliable therapeutic biomarkers of GBM, warrant further exploration.

Nuclear receptor‐binding protein 1 (NRBP1) (also known as pseudokinase) shares structural similarities with kinases but lack kinase activity. Contrary to the previous assumption that pseudokinases function solely as redundant kinases, several pseudokinases have been shown to play significant roles in physiological processes and, in some cases, exhibit kinase‐like activity, even in the absence of a conserved kinase domain sequence.^7^ They can regulate cellular signaling by acting as scaffolds, anchors, or allosteric modulators.^8^, ^9^ Additionally, pseudokinases are crucial players in cancer development and metastasis.^10^, ^11^ NRBP1 situated at the 2p23 locus, and encodes a remarkably conserved adaptor protein known for its widespread expression.^12^ NRBP1 with structural parallels to eukaryotic kinases, has the potential to engage in various cellular processes.^13^ Notably, it can interact with several critical transcription factors, including activated Rac3, the MLF1 oncoprotein, and JAB1, thus establishing its pivotal role in cellular functions.^14^

In the past decade, NRBP1 has been found to play different roles in cancer development. As an example, NRBP1 demonstrated anti‐tumor properties in intestinal cells, as evidenced by the heightened susceptibility of NRBP1‐null mice to intestinal malignancies.^14^ In addition, the protein has been shown to promote prostate and bladder cancer progression,^15^, ^16^ while inhibiting the development of lung adenocarcinomas and colorectal cancers.^14^, ^17^ There is still ongoing discussion about the involvement of NRBP1 in breast cancer. Recent research has confirmed that NRBP1 activates Rac1/Cdc42 through P‐Rex1, promoting oncogenic signal transduction in triple‐negative breast cancer.^18^ Previously, an initial investigation had documented NRBP1's suppressing effect on breast cancer cell proliferation.^19^ Nevertheless, the functional implications and expression patterns of NRBP1 in gliomas remain enigmatic.

Here, we present findings showcasing significantly elevated NRBP1 mRNA levels in GBM tissues when compared to both low‐grade glioma and normal tissues, thereby indicating a more unfavorable prognosis. Through our manipulation of NRBP1 expression levels in GBM cell lines, we have unearthed its pivotal role in fostering malignant phenotypes and driving EMT within the context of GBM. This modulation is achieved by orchestrating PI3K/Akt activation, a mechanism observed both in vitro and in vivo. As a result, we posit that NRBP1 operates as an oncogene within the domain of GBM. Moreover, given its potential to function as a prognostic indicator and novel target for GBM treatment, our findings hold significant implications for advancing therapeutic strategies in GBM management.

## 2 MATERIALS AND METHODS

### 2.1 Bioinformatical analyses

The bioinformatics analysis utilized data sourced from the Chinese Glioma Genome Atlas (CGGA; http://www.cgga.org.cn/) and The Cancer Genome Atlas (TCGA; https://cancergenome.nih.gov/). Using GEPIA (http://gepia.cancer‐pku.cn), we examined NRBP1 mRNA expression levels in gliomas with CNS WHO grade, 1p/19q co‐deletion status, IDH1 mutation status, overall survival (OS), and disease‐free survival (DFS). NRBP1 related differentially expressed genes (DEGs, *p* < 0.05, |logFC| >2) were obtained from TCGA and used for further ontological analyses. To assess the biological relevance of DEGs, Gene Ontology (GO) enrichment analysis was conducted to determine the enrichment of NRBP1 with respect to biological processes, molecular functions, and cellular components. Additionally, Kyoto Encyclopedia of Genes and Genomes (KEGG) enrichment analysis of DEGs was undertaken to elucidate the signaling pathways associated with NRBP1. These analyses were carried out using the R software packages “clusterProfiler” and “enrichplot.”

### 2.2 Clinical tissue sample collection

From January 2021 to June 2022, 10 normal tissue samples and 20 low‐ and 20 high‐grade glioma samples were harvested from the First Affiliated Hospital of USTC. The recently obtained samples were immediately placed in deep freeze at a temperature of −80°C. In accordance with the WHO Classification of CNS Tumors (5th Edition), two pathologists classified 305 surgically removed paraffin‐embedded intracranial glioma tissues collected between October 2019 and July 2022. The sample set comprised 30 cases of pilocytic astrocytomas (classified as CNS WHO grade 1), 34 instances of IDH‐mutant astrocytomas (classified as CNS WHO grade 2), 30 cases of IDH‐mutant astrocytomas (classified as CNS WHO grade 3), 47 cases of oligodendrogliomas (classified as CNS WHO grade 2), 32 cases of oligodendrogliomas (classified as CNS WHO grade 3), and 132 cases of IDH wild‐type GBMs (classified as CNS WHO grade 4). Patient clinical data were systematically collected and summarized. Kaplan–Meier survival analysis was conducted to evaluate the prognostic significance of NRBP1 in 132 GBM patients, with follow‐up data available for 115 patients. The experimental procedures in this study received ethical approval from the hospital's ethics committee (approval number: 2022‐RE‐298).

### 2.3 Quantitative real‐time PCR (qRT‐PCR) assay

The TRIzol reagent (Thermo Fisher Scientific, USA) was used to conduct total RNA extraction from human glioma tissue samples. We used a TaKaRa reverse transcription kit (Japan) to generate cDNA. The cDNA was then used in qRT‐PCR assays to quantify the relative levels of NRBP1 mRNA, based on the2^−△△Ct^ algorithm. The SYBR Green Real‐Time PCR Master Mix (Roche, Switzerland) was used for the qRT‐PCR assay, and all reactions were performed on the 7500 Fast Real‐Time PCR System. The primers employed in these assays included β‐actin with a sense primer: 5′‐CATGTACGTTGCTATCCAGGC‐3′ and antisense primer: 5′‐CTCCTTAATGTCACGCACGAT‐3′, as well as NRBP1 with a sense primer: 5′‐GGACTCATCAAGATTGGCTCTG‐3′ and antisense primer: 5′‐TCTTCTGCTCTTCTCGACAAGT‐3′. *β*‐actin was used as the internal reference gene.

### 2.4 Immunohistochemistry and staining

The specimens of intracranial glioma preserved in paraffin were cut into sections with a thickness of 4 μm. The sections were taken out from xylene and placed in different concentrations of alcohol for rehydration. Antigen fixation was carried out using a citric acid buffer (pH 6.0), and endogenous peroxidase activity was blocked by washing the slides with phosphate‐buffered saline (PBS). Then, the sections were subjected to overnight incubation in an NRBP1 primary antibody solution (GeneTex, Beijing, China) at 1:250 dilution at 4°C, followed by incubation with horseradish peroxidase‐labeled goat anti‐mouse IgG antibody (Beyotime, Beijing, China) for 30 min at ambient temperature. The staining level was evaluated by the percentage of tumor cells that tested positive, and graded as follows 0% (score 0), 1%–5% (score 1), 6%–25% (score 2), 26%–75% (score 3), and 76%–100% (score 4). For staining intensity, scores of 0–3 were assigned to negatively, weakly, mediumly, and strongly stained samples, respectively. The scores, which were determined by multiplying the staining extent score by the staining intensity score, ranged from 0 to 12. To perform the statistical analysis on cytoplasmic expression, a score of 0–4 represented low expression, whereas a score of 5–12 indicated high expression.

### 2.5 Cell culture

We purchased human GBM cell lines A172, SHG44, U87, and U251 and the normal astrocyte cell line HA1800 from the Institute of Biophysics, Chinese Academy of Sciences (Shanghai, China). Cultured cells were incubated at 37°C in Dulbecco's modified Eagle's medium supplemented with 10% fetal calf serum and 1% antibiotics (streptomycin and penicillin, Amimed, Switzerland) in a humidified incubator with a 5% CO_2_ atmosphere.

### 2.6 Western blotting

Cell lysis was induced by incubating the samples for 30 min in ice‐cold radioimmunoprecipitation assay lysis buffer. The bicinchoninic acid assay (Beyotime, Beijing, China) was used to determine the total protein concentrations. All protein samples underwent sodium dodecyl sulfate‐polyacrylamide gel electrophoresis before being electrotransferred to a polyvinylidene difluoride membrane. The blots were immersed for 60 min in 5% BSA in Tris‐buffered saline with Tween‐20 (TBST, pH 7.0) and then subjected to overnight incubation (at 4°C) with primary antibodies, including those against NRBP1 (1:1000; GeneTex, China), Bcl‐2 (Product #13–8800), Bax (Product #14–6997‐82), PI3K (Product #MA1‐74183), p‐PI3K (Tyr458, Tyr199, Product #PA5‐17387), Akt (Product #MA5‐14916), and p‐Akt (Ser473, Product #700392) (1:1000; Thermo Fisher Scientific, USA), and N‐cadherin (Product #13116, Cell Signaling Technology, USA), E‐cadherin (Product #3195, Cell Signaling Technology, USA), MMP‐7 (Product #71931) (1:1000; Cell Signaling Technology, USA), and *β*‐actin (1:5000; Cell Signaling Technology, USA). The blots were visualized by HRP‐labeled goat anti‐mouse or goat anti‐rabbit IgG (1:5000; CoWin Biosciences, China) using enhanced chemiluminescence kits (Millipore, USA). *β*‐actin bands served as the loading control.

### 2.7 Lentiviral infection

We bought the lentivirus plasmid from Beyotime Biotechnology located in Shanghai, China. For lentiviral infection, 1 × 10^6^ U87 cells were grown in the presence of 4 μL lentivirus with NRBP1 for 12 h, and lentivirus without NRBP1 was the negative control. After being cultured for 48 h at 37°C, selection of the stable cell lines was performed in the presence of 4 μg/mL puromycin. The cell status was observed every day, the new medium containing puromycin was replaced in time. After a period of 5 days, the cells that had survived were examined and stable cell lines were effectively established. We designed three shRNAs to target NRBP1 mRNA, with the sequences as follows: shNRBP1‐1, GACCTTGAACAAGTTCAATTT; shNRBP1‐2, GCAATGGAGAGTCCTCATATG; and shNRBP1‐3, CCAACACATGATCCCAGAGAA. After the stable cell lines were successfully established, transfection efficiency detected using Western Blot after 48 h. For subsequent experiments, the two groups of cells with superior transfection efficiency were chosen. NRBP1‐overexpressing (pcDNA‐NRBP1) and empty plasmids were bought from GenePharma (Shanghai, China) and transfected into cells that maintained in Opti‐MEM by utilizing Lipofectamine 2000. We evaluated transfection efficiency by collecting the cells 48 h after transfection. It is worth noting that NRBP1 is categorized as a “common essential” gene by DepMap (www.depmap.org), indicating that knocking down or overexpressing this gene may potentially have adverse effects on tumor cells.

### 2.8 Cell proliferation assay and colony formation

Cell proliferation capacity was evaluated using Cell Counting Kit‐8 (CCK‐8) assays. In brief, cells in logarithmic growth phase were inoculated at a density of 3000 cells per well into 96‐well plates and subsequently incubated for 0, 24, 48, 72, or 96 h. The CCK‐8 reagent (Dojindo Laboratories, Kyushu Island, Japan) was added to each well and incubated for 4 h. Afterwards, the optical density at a wavelength of 450 nm was measured using a Synergy H1 microplate reader from BioTek, located in the United States.

5 × 10^2^ cells were maintained in one well of a 6‐well plate, with the medium refreshed once on the fourth day of culture for 2 weeks. Cloned cells were fixed in a 4% paraformaldehyde solution for a duration of 15 min, followed by desiccation, and then underwent crystal violet staining. Six randomly picked fields were observed under an Olympus optical microscope for colony number counting (colonies with ≥50 cells were considered a single clone).

### 2.9 Wound healing assay

2 × 10^6^ cells were allowed to proliferate for 12 h in each well of a six well cell culture plate to achieve 90%–100% confluence. Later, scratching wounds were created using a sterilized pipette tip. After two PBS washes, the cells were maintained in a culture medium without serum for evaluating wound closure. A phase contrast microscope (×100) was used to visualize the cultured cells immediately and 24 h after the wounds were created to assess the percentage of wound healing that was determined as [(Ai− At)/Ai] × 100, in which Ai is the wound area determined immediately, and At denotes the wound area after cell culture.

### 2.10 Investigation on the migratory and invasive capabilities of glioma cells

The migratory and invasive abilities of glioma cells were evaluated using Transwell assays, for which the Matrigel was applied in the upper chamber only in the cell invasion analysis. 2 × 10^5^ cells that underwent starvation treatment were inoculated into an even distribution in the upper chamber containing culture medium without serum. In the lower chamber, cell culture medium plus 10% FBS, which served as a chemoattractant, was added. The Transwell chambers were then incubated for 24 h at 37°C, after which cells in the lower chamber were subjected to fixation and crystal violet (1%) staining. Cell counting was conducted using five different fields of view.

### 2.11 Cell apoptosis assay

A TUNEL kit (Beyotime, China) was used to assess DNA fragmentation, which is a hallmark of apoptosis, in accordance with the provider's protocol. A total of 2 × 10^6^ cells were seeded on glass slides within individual wells of a 6‐well plate and cultured for a duration of 3–4 days. Subsequently, the cells were mounted in a fluorescence‐preserving solution and examined using an Olympus fluorescence microscope (Leica, Weztlar, Germany). We randomly picked five high‐power fields (×200 magnification) to determine the numbers of TUNEL‐ and Hoechst‐positive cells, the proportion of which reflects the number of apoptotic cells.

### 2.12 AKT phosphorylation assay

U251 cells overexpressing NRBP1 (pcDNA‐NRBP1) were incubated with 10 μM MK‐2206 (Selleck, Houston, USA) for 48 h, and U87 cells with NRBP1 knockdown (sh‐NRBP1) were incubated with 10 μg/mL SC79 (Selleck, Houston, USA) for 48 h.

MK‐2206 or SC79 is dissolved in DMSO and diluted with culture medium to the above concentration before experimental use. U251 and U251 (pcDNA‐NRBP1) cells that were not incubated with MK‐2206 served as controls, whereas U87 and U87 (sh‐NRBP1) cells that were not incubated with SC79 were used as controls. After that, we carried out CCK‐8 and Transwell assay to evaluate cell proliferation and invasion. After the cells were incubated with MK2206 or SC79, the expression levels of NRBP1, p‐Akt, Akt, and EMT proteins in each group were detected by Western Blot.

### 2.13 Xenograft models

Female BABL/c nude mice (6‐week‐old, SLAC laboratory animal Center, Shanghai, China) were randomly divided into four groups (*n* = 5 to each group): U87‐sh‐control, U87‐sh‐NRBP1, U251‐Control, and U251‐pcDNA‐NRBP1. Each mouse was injected with 1 × 10^7^ cells subcutaneously into the right flank. In U87‐sh‐NRBP1 group, one transplanted tumor had never grown up. In U251‐Control and U251‐pcDNA‐NRBP1 groups, one nude mouse was bitten to death by another nude mouse respectively, none of them died from transplanted tumor. Tumor size was measured on length (a) and width (b) by using a caliper every 7 days, tumor volume was calculated with the equation: volume = (a × b^2^)/2. After 28 days, tumors were removed, weighted and froze at −80°C or fixed in 4% neutral formaldehyde for further analysis. All experimental procedures involving animals received prior approval from the Animal Experiment Administration Committee of The First Affiliated Hospital of USTC (202402031527000321741).

### 2.14 Statistical analysis

We employed GraphPad Prism 8.0 (GraphPad Software, USA) for analysis of statistical data. Data from three assays performed in triplicate are displayed as mean ± SEM. The Kaplan–Meier method was utilized to construct survival curves for both DFS and OS, with intergroup disparities assessed through the log‐rank test. A statistical analysis was conducted using either one‐way ANOVA or a two‐tailed student's *t*‐test to determine if the observed differences were statistically significant. Values with *: *p* < 0.05, **: *p* < 0.01, and ***: *p* < 0.001 are indicative of statistical significance.

## 3 RESULTS

### 3.1 NRBP1 expression in glioma

**FIGURE 1:**
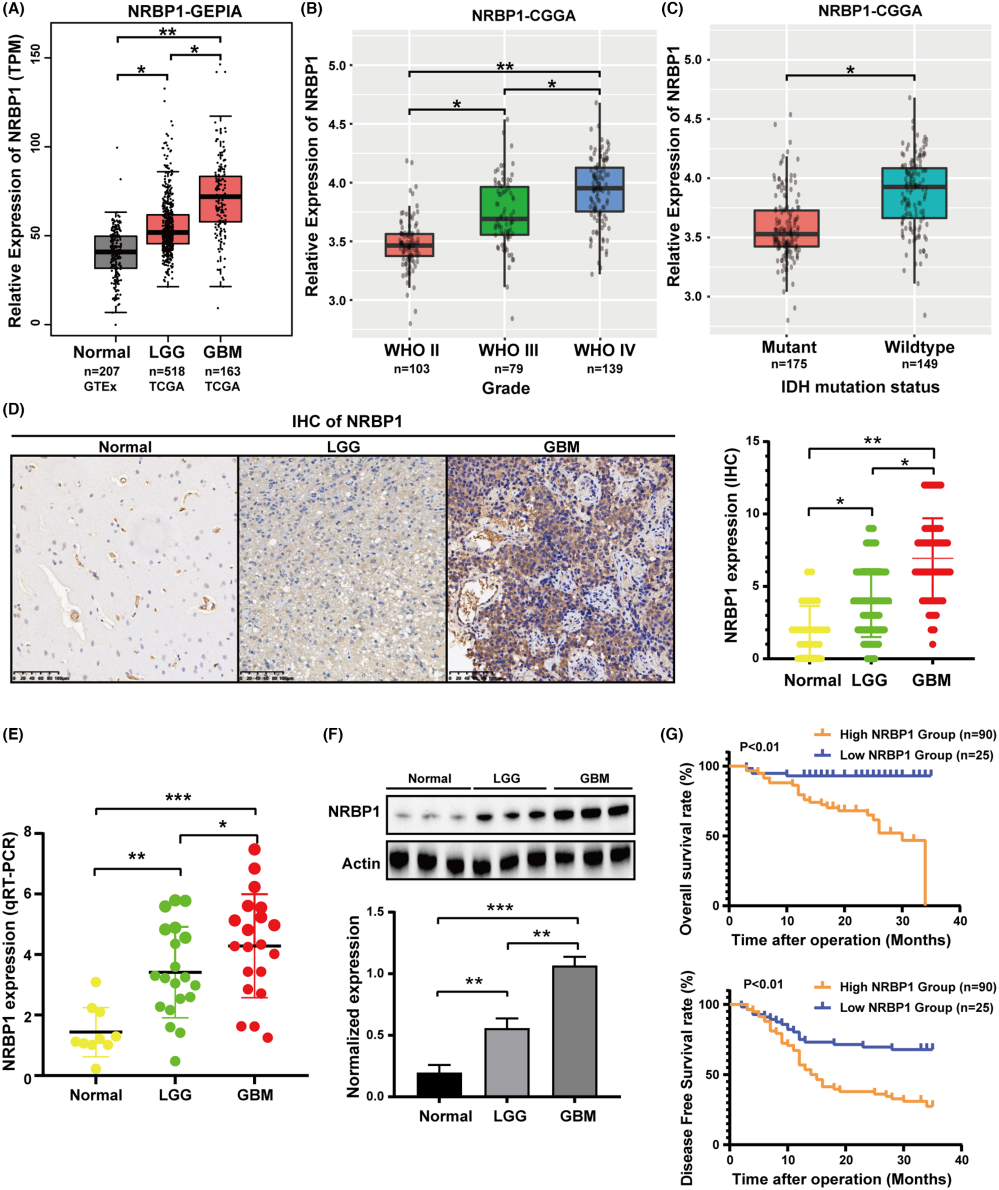
Nuclear receptor‐binding protein 1 (NRBP1) expression in glioma. NRBP1 mRNA levels of glioma and normal tissues obtained from TCGA (A) and CGGA (B, C) Correlations between NRBP1 and IDH status in gliomas determined using CGGA. (D) Representative IHC staining images for NRBP1 in normal tissues, IDH‐mutant astrocytomas (CNS WHO grade 2) and Glioblastoma (GBM). (E) qRT‐PCR conducted to measure relative expression levels of NRBP1 in different grade glioma and normal tissue samples. (F) Western blotting conducted to measure protein expression levels of NRBP1 in different grade glioma and normal tissue samples. (G) High NRBP1 expression had positive correlations with reduced OS and DFS as determined through IHC staining.

To explore the NRBP1 function in gliomas, we compared NRBP1 mRNA expression in gliomas and normal tissues using the TCGA/GEPIA and CGGA datasets. Significantly upregulated mRNA expression of NRBP1 was observed in GBM tissues compared with that in normal or low‐grade glioma tissues (Figure 1A). The expression showed a positive correlation with the grade of glioma (Figure 1B). Furthermore, the mRNA level of NRBP1 was negatively correlated with the IDH mutant status (Figure 1C) and 1p/19q co‐deletion. Immunohistochemistry identified significantly higher positive staining intensity of NRBP1 in GBM tissues than in normal or low‐grade glioma tissues (Figure 1D). Only 21 out of 111 (18.9%) of the low‐grade glioma showed high expression of NRBP1, whereas 125 out of 194 (64.4%) of the high‐grade glioma showed high expression of NRBP1. There was a greater expression of NRBP1 mRNA and protein in high‐grade glioma tissues compared to normal or low‐grade glioma tissues (Figure 1E,F). The clinicopathological features of the 305 patients with gliomas are presented in Table 1. Univariate analysis demonstrated significant correlations between NRBP1 expression levels and age, WHO grade, IDH mutant status, and 1p/19q co‐deletion (Table 1). One hundred and fifteen patients were tested at the median follow‐up of 20 months (range:13–34), the median OS and DFS of patients with high NRBP1 expression was significantly shorter than that of patients with low NRBP1 expression (Figure 1G). These results are consistent with those available in the TCGA database, suggesting that high NRBP1 expression predicts poor survival in GBM.

**TABLE 1:** Association of NRBP1 with clinicopathological characteristics.

Characteristics	Patient number	Low expression	High expression	*p*‐value
Sex				
Male	171	87 (50.9%)	84 (49.1%)	
Female	134	72 (53.7%)	62 (46.3%)	0.6204
Age				
<55	137	100 (73.0%)	37 (27.0%)	
≥55	168	59 (35.1%)	109 (64.9%)	<0.05
WHO grade				
1 + 2	111	90 (81.1%)	21 (18.9%)	
3 + 4	194	69 (35.6%)	125 (64.4%)	<0.05
IDH				
Mutant	143	96 (67.1%)	47 (32.9%)	
Wild	162	63 (38.9%)	99 (61.1%)	<0.05
1p/19q				
Co‐deletion	79	58 (73.4%)	21 (26.6%)	
No co‐deletion	226	101 (44.7%)	125 (55.3%)	<0.05

### 3.2 NRBP1 silencing suppressed cell proliferation, invasion, and migration and induced apoptosis

**FIGURE 2:**
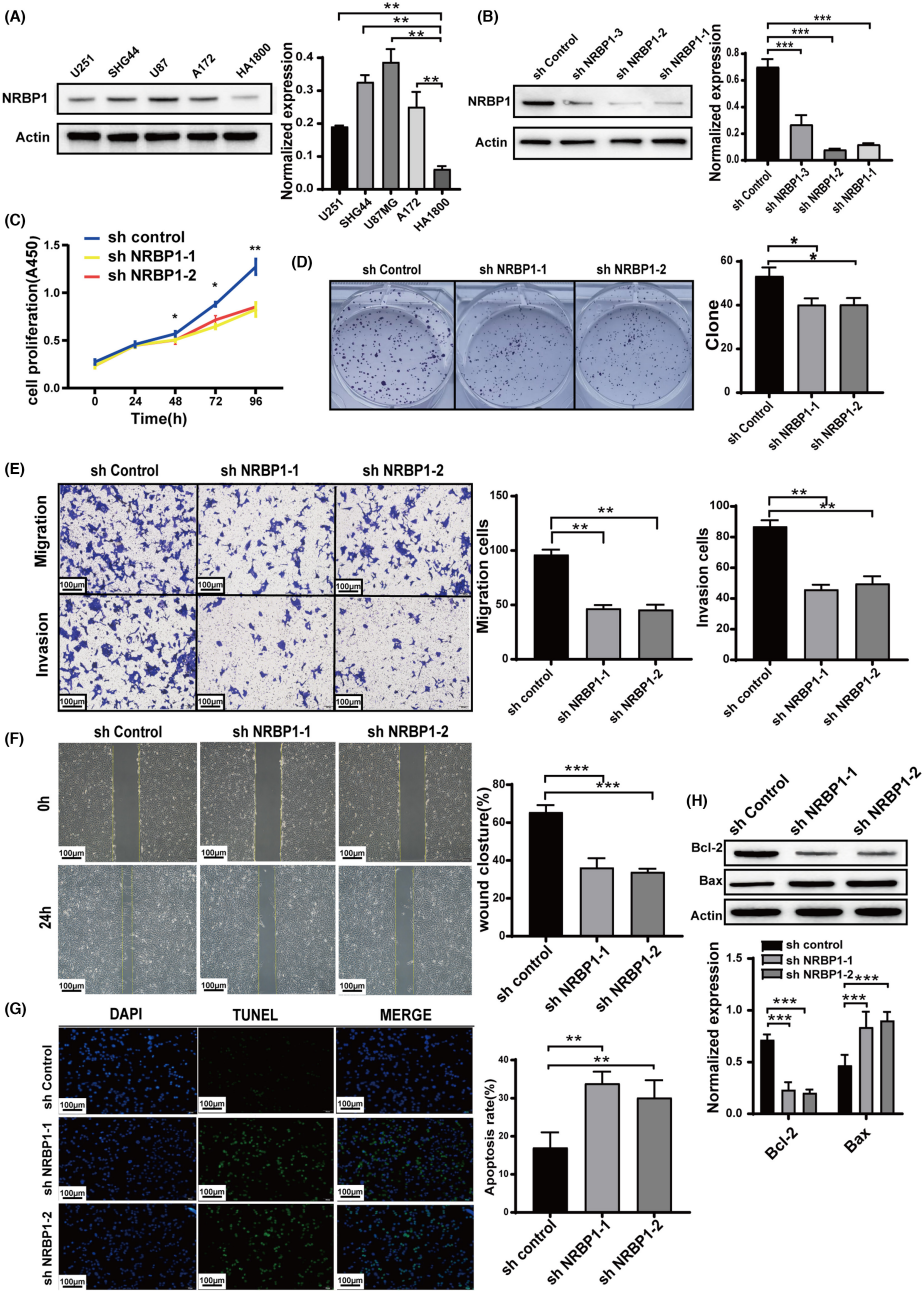
Downregulation of Nuclear receptor‐binding protein 1 (NRBP1) Suppresses Cell Proliferation, Invasion, and Migration and Induces Apoptosis. (A) Immunoblotting analysis of NRBP1 expression in Glioblastoma (GBM) cell lines (U251, SHG44, A172, and U87MG) and a normal astrocyte cell line (HA1800). (B) Western blot results illustrating NRBP1 levels after transfection with sh‐NRBP1‐1, sh‐NRBP1‐2, and sh‐NRBP1‐3. (C, D) CCK‐8 and colony formation assays conducted to assess cell proliferation in sh‐NRBP1‐transfected cells. (E) Transwell assay used to compare cell migration and invasion abilities in sh‐NRBP1‐transfected cells. (F) Wound healing assay to evaluate cell migration ability in sh‐NRBP1‐transfected cells. (G, H) TUNEL staining and western blotting for the assessment of cell apoptosis. *β*‐Actin was employed as the loading control in the study.

The biological roles of NRBP1 in GBM were analyzed by measuring NRBP1 expression in GBM cell lines U251, SHG44, U87, and A172 and a noncancerous astrocyte cell line HA1800. Glioma cells showed significantly higher NRBP1 expression than HA1800 cells (Figure 2A). U87 cells with the highest expression and U251 cells with the lowest expression were selected for NRBP1 knockdown and overexpression assays, respectively. Next, we transfected lentivirus particles packaged with three different shRNAs targeting NBRP1, which resulted in significantly reduced NRBP1 protein expression in U87 cells (Figure 2B). We selected shNRBP1‐1 and shNRBP1‐2 for cell function experiments because of their high knockdown efficiency. The CCK8 assay results were used to generate growth curves for shNRBP1‐1, shNRBP1‐2, and control cells. The curves showed a significant suppression of cell growth in both shNRBP1‐transfected cell lines (Figure 2C). The inhibition of cell growth caused by the downregulation of NRBP1 was additionally validated through the formation of colonies (Figure 2D). The sh‐NRBP1 groups showed a significant reduction in cell invasion and migration ability when compared to the control, as indicated by the Transwell and wound healing assays (Figure 2E,F). TUNEL staining and western blotting revealed that the sh‐NRBP1 groups exhibited significantly increased cell apoptosis than control cells (Figure 2G), demonstrated decreased Bcl‐2 levels and increased Bax levels (Figure 2H).

### 3.3 NRBP1 overexpression induced cell proliferation, invasion, and migration and suppressed apoptosis

**FIGURE 3:**
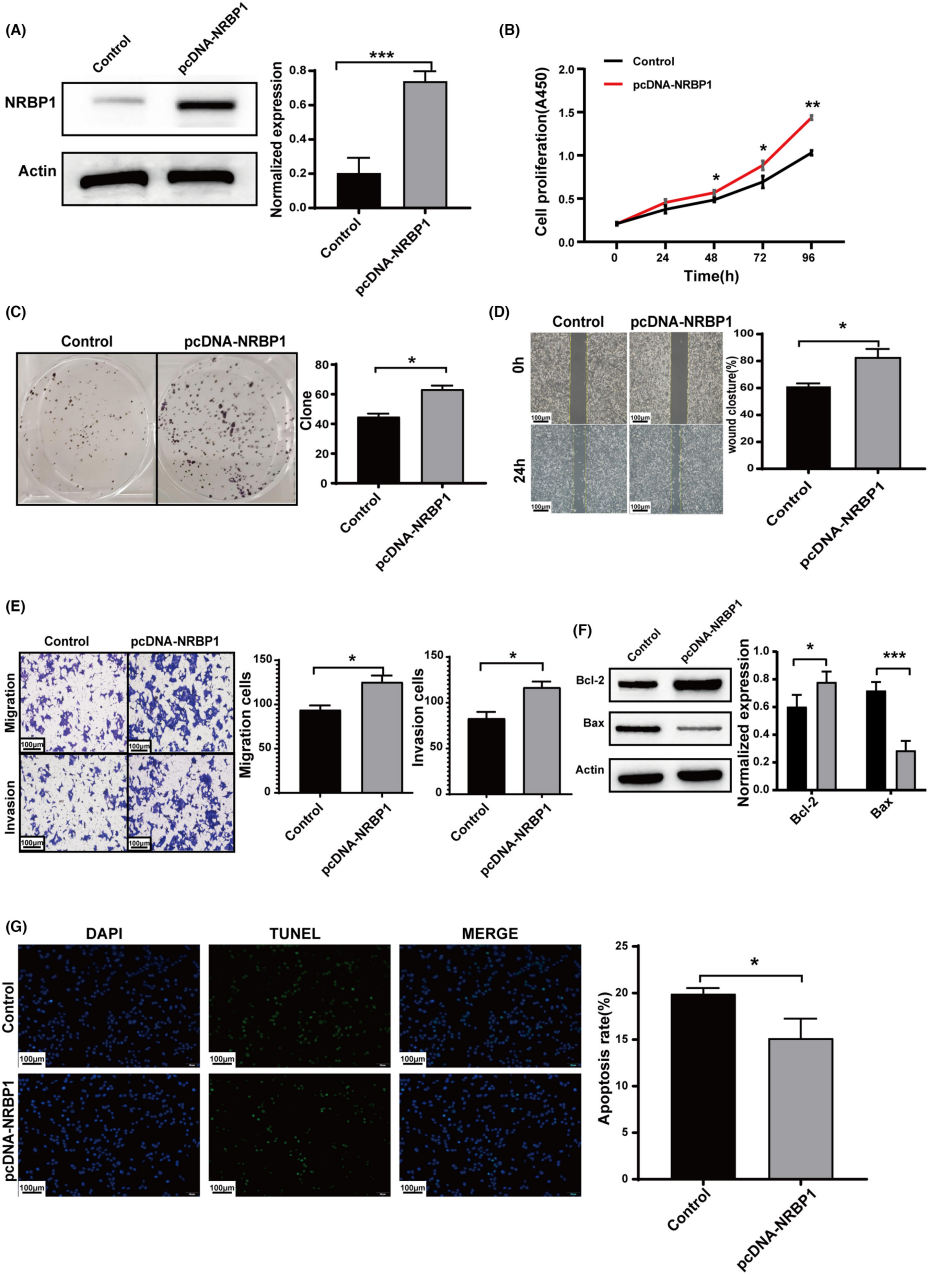
Enhanced Nuclear receptor‐binding protein 1 (NRBP1) expression facilitates cell proliferation, invasion, and migration while reducing cell apoptosis. (A) Immunoblotting analysis confirms the overexpression of NRBP1 in the U251 cell line. (B, C) CCK‐8 and colony formation assays were performed to quantify cell proliferation in pcDNA‐NRBP1‐transfected cells. (D) Wound healing assay was conducted to evaluate the migratory potential of pcDNA‐NRBP1‐transfected cells. (E) Transwell assay was used to compare the invasive and migratory capacities of pcDNA‐NRBP1‐transfected cells. (F, G) Western blotting and TUNEL staining were performed to assess cell apoptosis. *β*‐Actin served as the loading control in the study.

To validate the function of NRBP1 in GBM cells, we conducted overexpression experiments on U251 cells. Immunoblotting conducted to evaluate NRBP1 overexpression in U251 cell line (Figure 3A). Based on the results from colony formation and CCK‐8 assays, it was evident that NRBP1 overexpression had a noticeable impact on U251 cell proliferation (Figure 3B,C). The wound healing assay showed a significantly increased wound closure area at 24 h in pcDNA‐NRBP1 cells compared to the negative control cells (Figure 3D). Consistent results were observed in Transwell experiments. The pcDNA‐NRBP1 group showed significantly increased cell invasion and migration abilities compared to the vector group (Figure 3E). The expression of Bax decreased and Bcl‐2 increased in pcDNA‐NRBP1 group compared with that in the vector group (Figure 3F). TUNEL staining and western blotting revealed significantly decreased cell apoptosis in the pcDNA‐NRBP1 group (Figure 3G). In vitro experiments have shown that NRBP1 actively enhances the malignant characteristics of GBM.

### 3.4 NRBP1 controlled the expression of EMT markers and triggered PI3K/Akt phosphorylation

**FIGURE 4:**
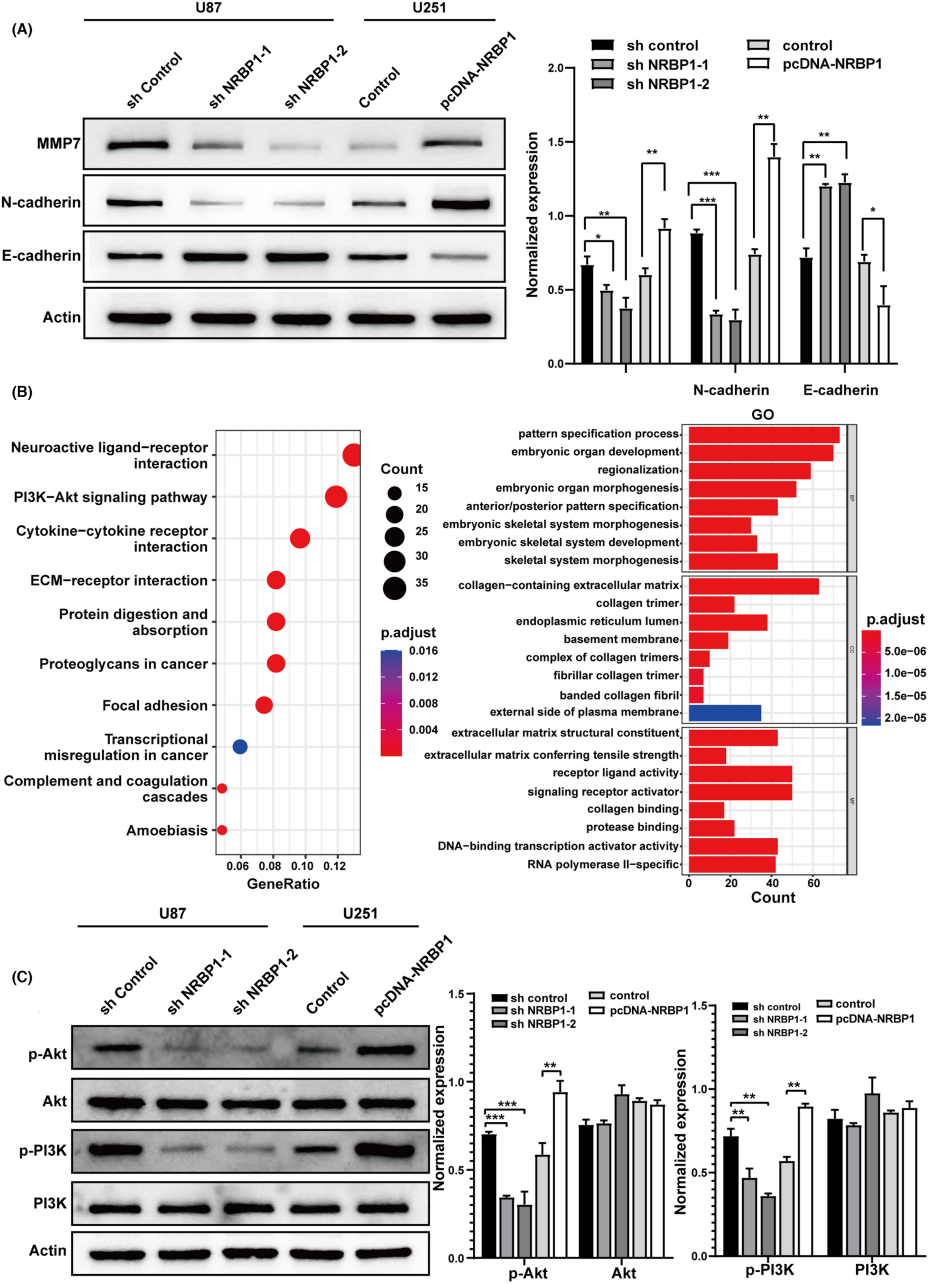
Nuclear receptor‐binding protein 1 (NRBP1) Regulated EMT marker expression and induced PI3K/Akt phosphorylation. (A) Western blotting with corresponding antibodies for determining EMT marker protein levels in shNRBP1‐ and pcDNA‐NRBP1‐treated U87 and U251 cells. (B) Enriched GO and KEGG term of NRBP1 molecular function indicated by dot plot. Dot size reflects the number of genes in NRBP1 that are associated with the GO and KEGG term, and the color indicates the adjusted *p*‐value. (C) Western blotting results of Akt, p‐Akt, PI3K, and p‐PI3K levels in shNRBP1‐ and pcDNA‐NRBP1‐treated U87 and U251 cells. *β*‐Actin served as the loading control in the study.

To assess the connection between NRBP1 and EMT, we measured the levels of MMP‐7, N‐cadherin, and E‐cadherin. These markers are associated with the EMT process. We examined their expression before and after NRBP1 knockdown and overexpression. NRBP1 knockdown augmented E‐cadherin expression but reduced N‐cadherin and MMP‐7 expression, whereas NRBP1 overexpression resulted in opposite effects (Figure 4A). The data confirms that NRBP1 triggers EMT in GBM cells. The analysis of GO enrichment and KEGG enrichment for the DEGs demonstrated that NRBP1 controls specific gene clusters involved in the PI3K/Akt signaling pathway. These regulatory effects were assessed using the “clusterProfiler” and “enrichplot” packages in the R programming environment (Figure 4B). To test the impact of NRBP1 on PI3K/Akt activation, we evaluated the protein levels of total and phosphorylated PI3K and AKT before and after NRBP1 knockdown and overexpression, which revealed that the phosphorylation levels of the two proteins were markedly higher than in the shNRBP1 group and lower than in the pcDNA‐NRBP1 group, while total PI3K and AKT expression remained unchanged (Figure 4C).

### 3.5 PI3K/Akt inhibitor or activator affected cell proliferation, invasion, and EMT via NRBP1

**FIGURE 5:**
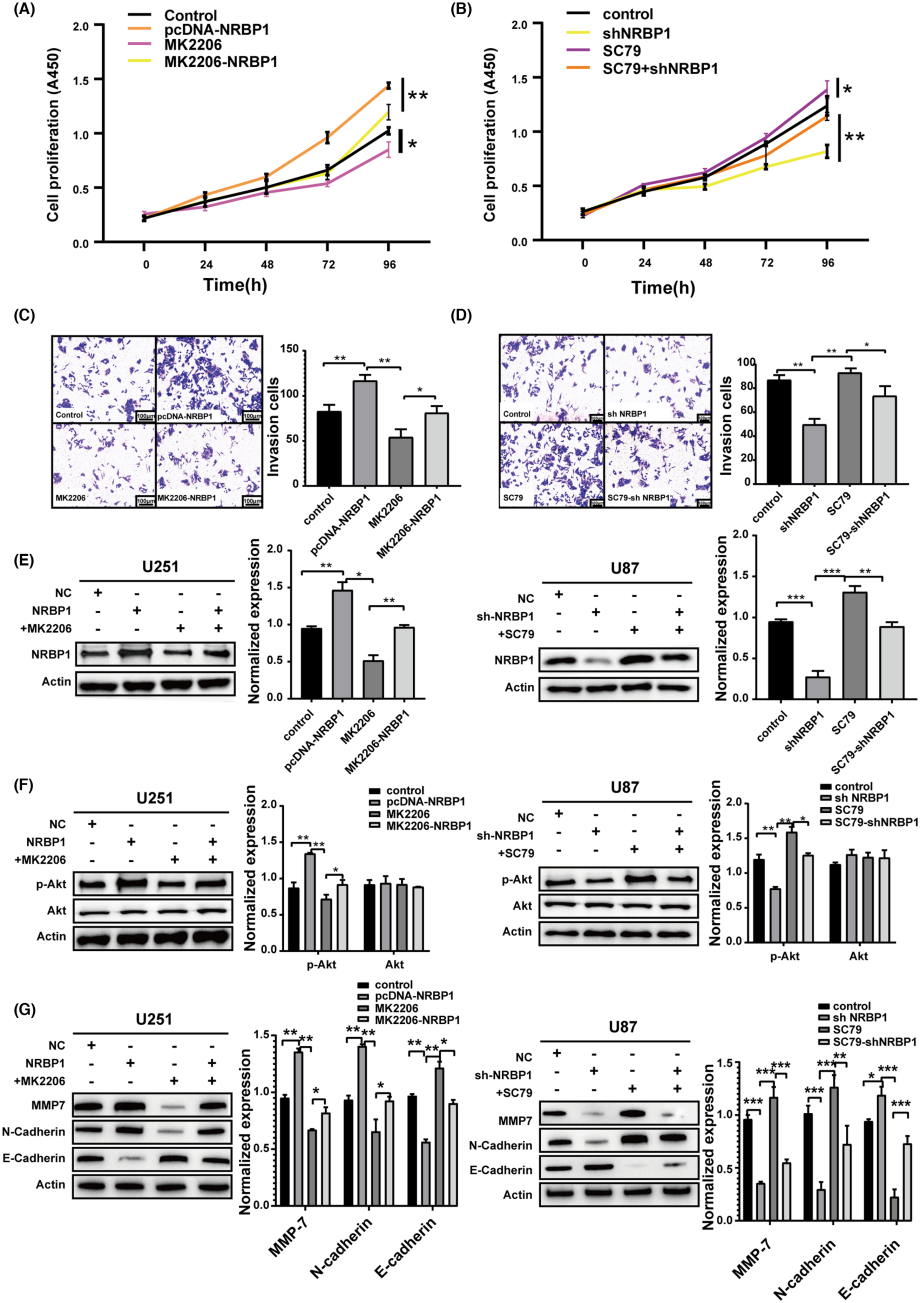
PI3K/Akt inhibitor or activator affected cell proliferation, invasion, and EMT by Nuclear receptor‐binding protein 1 (NRBP1). (A, B) CCK‐8 analysis for estimating cell proliferation in pcDNA‐NRBP1‐ or shNRBP1‐transfected cells with AKT inhibitor MK‐2206 or AKT activator SC79. (C, D) Transwell assay comparing the invasion ability of pcDNA‐NRBP1‐ or shNRBP1‐transfected cells added with AKT inhibitor MK‐2206 or AKT activator SC79. (E) Western blotting results of NRBP1 levels in shNRBP1‐ or pcDNA‐NRBP1‐treated U87 and U251 cells added with AKT inhibitor MK‐2206 or AKT activator SC79. (F) Western blotting results of Akt and p‐Akt levels in shNRBP1‐ or pcDNA‐NRBP1‐treated U87 and U251 cells added with AKT inhibitor MK‐2206 or AKT activator SC79. (G) Western blotting with corresponding antibodies showing the expression levels of EMT markers in shNRBP1‐ or pcDNA‐NRBP1‐treated U87 and U251 cells added with AKT inhibitor MK‐2206 or AKT activator SC79. *β*‐Actin served as the loading control in the study.

To investigate whether the impact of NRBP1 silencing or overexpression on GBM occurred via the PI3K/Akt signaling cascade, U251 cells overexpressing NRBP1 (pcDNA‐NRBP1) were treated with 10 μM AKT inhibitor MK‐2206, and U87 cells with NRBP1 knockdown (sh‐NRBP1) were incubated with 10 μg/mL AKT activator SC79. Notably, MK‐2206, an inhibitor against AKT, suppressed the proliferation and invasion of the pcDNA‐NRBP1 group, whereas the AKT activator SC79 promoted this in the sh‐NRBP1 group (Figure 5A–D). After treated with the MK‐2206, the expression of NRBP1 in U251 was downregulated, and MK‐2206 could inhibit the expression of NRBP1 in the pcDNA‐NRBP1 group. Similarly, in the SC79‐treated group, the expression of NRBP1 was upregulated, and SC79 could upregulate the expression of NRBP1 in the shNRBP1 group (Figure 5E). After treated with MK2206 or SC79, the levels of p‐Akt protein also were downregulated or upregulated, while the total AKT levels remained unchanged (Figure 5F). Moreover, MK‐2206 significantly inhibited N‐cadherin and MMP‐7 expression but upregulated E‐cadherin expression in the pcDNA‐NRBP1 group. The sh‐NRBP1 group showed a reversal of this effect when treated with SC79 (Figure 5G). These results reveal the possible involvement of NRBP1 in GBM progression via inducing EMT, which is triggered by AKT phosphorylation.

### 3.6 The cell function of NRBP1 in vivo

**FIGURE 6:**
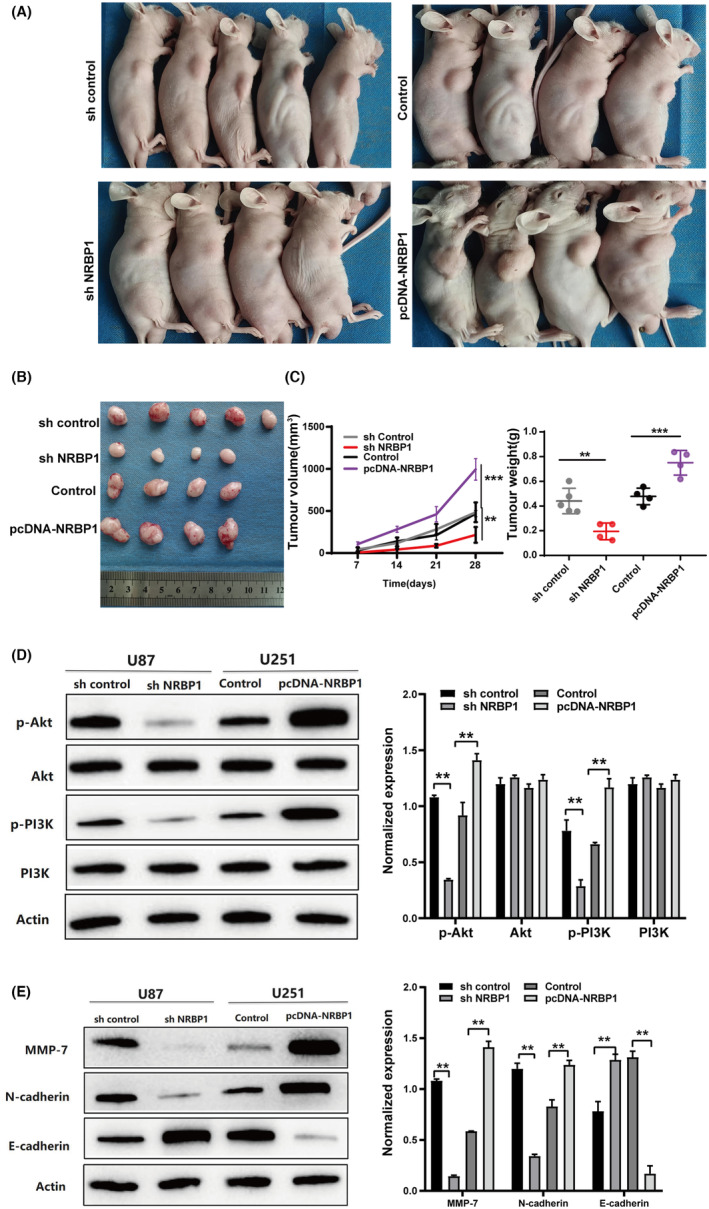
The function of Nuclear receptor‐binding protein 1 (NRBP1) in vivo. (A) Photographs of tumor bearing nude mice inoculated with U87‐sh‐control, U87‐sh‐NRBP1, U251‐Control, and U251‐pcDNA‐NRBP1 cells. (B) Tumors tissues isolated from the above four groups of nude mice. (C) Tumor volumes and weights of four groups in nude mice. (D) Western blotting results of Akt, p‐Akt, PI3K, and p‐PI3K levels in shNRBP1‐ and pcDNA‐NRBP1‐treated U87 and U251 cells in vivo. (E) Western blotting with corresponding antibodies for determining EMT marker protein levels in shNRBP1‐ and pcDNA‐NRBP1‐treated U87 and U251 cells in vivo. *β*‐Actin served as the loading control in the study.

To investigate whether NRBP1 influenced the tumor growth in vivo, xenograft mouse models were established. U87‐sh‐control, U87‐sh‐NRBP1, U251‐Control, and U251‐pcDNA‐NRBP1 cells were injected subcutaneously into BABL/c nude mice, tumor volume was measured at 7‐day intervals. The tumor growth in the U87‐sh‐NRBP1 group was slower than that of the U87‐sh‐control group, and faster in the U251‐pcDNA‐NRBP1 group than the U251‐Control group (Figure 6A–C). These results were consistent with the cell proliferation assay in vitro, suggesting that NRBP1 could play a role in glioma tumorigenesis in vivo. To further explore the mechanism of NRBP1 tumorigenesis in glioma, Western blotting analysis were performed. The phosphorylation levels of PI3K and AKT protein were markedly higher than that of sh‐NRBP1 group and lower than that of pcDNA‐NRBP1 group, while total PI3K and AKT expression remained unchanged (Figure 6D). Also, NRBP1 knockdown augmented E‐cadherin expression but reduced N‐cadherin and MMP‐7 expression, whereas NRBP1 overexpression resulted in opposite effects (Figure 6E).

## 4 DISCUSSION

In GBM, treatment failure and recurrence are caused by the highly malignant phenotype of GBM cells invading the surrounding brain parenchyma.^20^, ^21^ Therefore, there is a need to elucidate the complicated pathology of GBM so as to obtain novel insights into accurate prognostic prediction that is crucial for designing more effective therapeutic strategies. The pseudokinase NRBP1 has recently been described to have an important role in cancers, but the function and expression of NRBP1 in GBM is unidentified.

In this context, we present preliminary findings regarding the expression of NRBP1 in clinical samples of glioma tissues as well as cultured GBM cell lines and transplanted tumor in nude mice. Through our examination of the TCGA and CGGA databases, we discovered unfavorable outcomes in patients exhibiting elevated NRBP1 expression levels. Based on our analysis of clinical cases and bioinformatics data, our findings suggest a correlation between elevated NRBP1 expression and a higher glioma grade. The incidence of high NRBP1 expression in GBM (73.5%) significantly surpasses its occurrence in pilocytic astrocytomas (15%), astrocytomas (40.6%), and oligodendrogliomas (26.6%). The grade of CNS tumors is commonly believed to exhibit a positive correlation with patients' age. Furthermore, the histological WHO grade has been found to be positively linked to prognosis, as indicated in previous studies.^22^ The outlook for IDH‐mutant glioma is much better than that of IDH wild‐type GBM.^23^ Therefore, in the 2021 5th edition of the WHO classification for CNS tumors, IDH‐mutant astrocytoma, graded as CNS grade 4, was defined as a separate category from GBM. In our study, we noted that NRBP1 expression levels were significantly correlated with age, WHO grade, IDH status, and 1p/19q co‐deletion in the univariate analysis, consistent with previous findings showing upregulated NRBP1 expression in diverse cancers, including prostate cancer, bladder cancers, and triple‐negative breast cancers.^13^, ^14^, ^18^ The negative correlation between NRBP1 and 1p/19q co‐deletion may be due to the requirement for IDH mutation in the diagnosis of oligodendroglioma, and 1p/19q co‐deletion is also one of the diagnostic criteria for oligodendroglioma.^24^ The prognostic significance of NRBP1 was assessed in 115 GBM patients showed that high NRBP1 expression was positively correlated with reduced OS and DFS. The findings implied that an elevated NRBP1 expression is indicative of unfavorable survival rates in GBM and contributes to the development of glioma tumors.

To confirm whether NRBP1 advances GBM progression, GBM cell lines with NRBP1 overexpression and knockdown were used to examine cell proliferation, migration, invasion, and apoptosis. We selected U87 cells with the highest expression for NRBP1 knockdown assay and U251 cells with the lowest expression for NRBP1 overexpression assay. U87 cells transfected with NRBP1 exhibited notable suppression of proliferation, invasion, and migration, and concomitantly induced apoptosis. When NRBP1 was overexpressed in U251 cells, a notable rise in cell proliferation, invasion, and migration was observed. To investigate whether NRBP1 influences the tumor growth in vivo, xenograft mouse models were established. U87‐sh‐control, U87‐sh‐NRBP1, U251‐Control, and U251‐pcDNA‐NRBP1 cells were injected. The U87‐sh‐NRBP1 group exhibited reduced average tumor volume and weight when compared to the U87‐sh‐control group. In contrast, the U251‐pcDNA‐NRBP1 group demonstrated elevated average tumor volume and weight compared to the U251‐Control group. These data indicate that NRBP1 promotes the malignant phenotype of GBMs in vitro and in vivo.

In terms of NRBP1 mechanism, we demonstrated that NRBP1 plays a critical role in promoting EMT through the activation of AKT for the first time. EMT plays a pivotal role in embryonic development^25^ and constitutes a significant molecular event in tumor invasion, including GBM.^26^ Our observations indicate that NRBP1 knockdown upregulates E‐cadherin expression while downregulating MMP‐7 and N‐cadherin expression, with the opposite effect seen in NRBP1 overexpression. Therefore, NRBP1 appears to modulate EMT in GBM cells. EMT is governed by multiple signaling pathways^27^ and is associated with oncogenic activation of the AKT protein kinase.^28^ The malignant phenotypes of GBM can be influenced by various molecular pathways, such as PI3K/Akt pathway, NF‐κB pathway, Hedgehog pathway, and RB/E2F pathway. The PI3K/Akt signaling pathway exerts influences on cell proliferation, invasion, and migration across various cancers, such as astrocytomas, melanomas, endometrial cancer, breast cancer, renal cancer, ovarian cancer, pulmonary cancer, and lymphoid cancer.^29^ GO enrichment and KEGG analyses revealed that NRBP1 regulates differentially expressed gene clusters implicated with the PI3K/Akt signaling cascade and extracellular matrix (ECM). We also verified whether NRBP1 expression is associated with Akt phosphorylation at the molecular level in vitro and in vivo. It identified that NRBP1 downregulation inhibited AKT and PI3K phosphorylation in U87 cells, which increased after NRBP1 overexpression in U251 cells. In addition, treatment with the specific inhibitor MK‐2206 or the activator SC79 reversed the effect of NRBP1 silencing or overexpression on GBM. The ECM, which undergoes significant changes during EMT, can activate the PI3K/AKT pathway through signaling molecules like integrins. This activation forms a positive feedback loop, enhancing AKT signaling and potentially promoting EMT progression. Our results strongly indicate NRBP1 involves regulating ECM dynamics or influencing the responsiveness of cells to ECM cues, thereby modulating the activation of AKT and subsequent EMT events.

Our study presents the inaugural report on the correlation between NRBP1 and the PI3K/Akt signaling pathway, along with EMT, within the context of GBM. This revelation introduces a fresh viewpoint into comprehending the molecular intricacies of this disease. These findings hold the potential to unveil the mechanisms responsible for the onset and progression of GBM, while also offering an avenue for the identification of novel therapeutic targets.

The findings of this study have to be seen in light of some limitations. The first is the limitted clinical data were collected, although we collected 305 cases of gliomas, GBM only accounts for one‐third, and not all GBM were be followed up, the time from surgical resection to follow‐up was not long enough. And this is a single center data. In the future, we hope to collect clinical data from multi‐centers. The second limitation concerns the more systemic mechanisms of NRBP1 promotes the malignant phenotypes of GBM by regulating PI3K/Akt activation. In future research, we aim to identify specific molecular targets that bind to NRBP1 in the PI3K/Akt signaling pathway. Further molecular identifying, including determination of the mechanism and function of NRBP1, is required to inform drug development programs.

## 5 CONCLUSION

NRBP1 overexpression was correlated with the progression and dismal clinical outcomes of GBM via inducing EMT which is activated through AKT phosphorylation.

## AUTHOR CONTRIBUTIONS

**Anli Zhang:** Conceptualization (equal); methodology (equal); software (equal); writing – original draft (lead). **Shichao Peng:** Conceptualization (equal); data curation (lead). **Sibai Sun:** Conceptualization (equal); methodology (equal). **Shan Ye:** Conceptualization (equal); methodology (equal). **Ye Zhao:** Funding acquisition (lead); investigation (equal); supervision (equal). **Qiang Wu:** Project administration (lead); supervision (lead).

## FUNDING INFORMATION

This work was supported by financial support from National Natural Science Foundation of China (Grant number 12275003).

## CONFLICT OF INTEREST STATEMENT

The authors declare that the research was conducted in the absence of any commercial or financial relationships that could be construed as a potential conflict of interest.

## ETHICS STATEMENT

Our study was approved by the Ethics Committee of The First Affiliated Hospital of USTC (No: 2022‐RE‐298). All experimental procedures involving animals received prior approval from the Animal Experiment Administration Committee of The First Affiliated Hospital of USTC (202402031527000321741).

## Data Availability

The original contributions presented in the study are included in the article material, further inquiries can be directed to the corresponding author.
